# fMRI Evidence for a Cortical Hierarchy of Pitch Pattern Processing

**DOI:** 10.1371/journal.pone.0001470

**Published:** 2008-01-30

**Authors:** Lauren Stewart, Tobias Overath, Jason D. Warren, Jessica M. Foxton, Timothy D. Griffiths

**Affiliations:** 1 Department of Psychology, Goldsmiths, University of London, London, United Kingdom; 2 Wellcome Trust Centre for Neuroimaging, Institute of Neurology, London, United Kingdom; 3 Auditory Group, Newcastle University Medical School, Newcastle upon Tyne, United Kingdom; 4 Dementia Research Centre, Institute of Neurology, Queen Square, London, United Kingdom; 5 INSERM, Unité 280, Centre Hospitalier Le Vinatier, Lyon, France; Ludwig Maximilians University Munich, Germany

## Abstract

Pitch patterns, such as melodies, consist of two levels of structure: a global level, comprising the pattern of ups and downs, or contour; and a local level, comprising the precise intervals that make up this contour. An influential neuropsychological model suggests that these two levels of processing are hierarchically linked, with processing of the global structure occurring within the right hemisphere in advance of local processing within the left. However, the predictions of this model and its anatomical basis have not been tested in neurologically normal individuals. The present study used fMRI and required participants to listen to consecutive pitch sequences while performing a same/different one-back task. Sequences, when different, either preserved (local) or violated (global) the contour of the sequence preceding them. When the activations for the local and global conditions were contrasted directly, additional activation was seen for local processing in right planum temporale and posterior superior temporal sulcus (pSTS). The presence of additional activation for local over global processing supports the hierarchical view that the global structure of a pitch sequence acts as a “framework” on which the local detail is subsequently hung. However, the lateralisation of activation seen in the present study, with global processing occurring in left pSTS and local processing occurring bilaterally, differed from that predicted by the neuroanatomical model. A re-examination of the individual lesion data on which the neuroanatomical model is based revealed that the lesion data equally well support the laterality scheme suggested by our data. While the present study supports the hierarchical view of local and global processing, there is an evident need for further research, both in patients and neurologically normal individuals, before an understanding of the functional lateralisation of local and global processing can be considered established.

## Introduction

Cognitive neuropsychological studies have demonstrated that pitch patterns, such as melodies, consist of two structural levels: the contour or pattern of ups and downs–synonymous with the ‘global’ level; and the precise intervals that make up this contour–synonymous with the ‘local’ level. Early behavioural support for this hierarchical model came from same/different tasks in which pairs of novel pitch sequences could differ at a local level, where contour is preserved, or at a global level, where the overall contour is violated [1]. Individuals can reach high levels of accuracy in the detection of both types of change. However, if the second sequence is shifted in overall pitch, individuals are unable to detect differences where the contour is preserved. The dependence of participants' accuracy on the presence or absence of a change in contour suggests that processing of contour provides a ‘scaffold’ on which the detail of the precise intervals are subsequently ‘hung’ (see [2]–[4] for further behavioural evidence of this model).

Evidence for the neuro-anatomical basis of this model has come from patient studies. Peretz [5] tested patients with heterogeneous left or right hemispheric damage (LHD or RHD, respectively) on tasks similar to those described above. Deficits in the detection of differences involving a contour violation always co-existed with deficits in the detection of differences where the contour was preserved. In contrast, selective deficits in discriminating melodies that shared the same contour were seen without accompanying deficits in discriminating melodies that differed in contour. Moreover, this pattern was associated with damage to different hemispheres: RHD patients were worse than normal control (NC) participants for the detection of both types of differences, while LHD patients performed significantly better for contour-violated than contour-preserved differences.

A similar pattern of results was found by Liégeois-Chauvel et al. [6] in patients with lesions confined to the temporal lobes. Lesions to right posterior temporal cortex were associated with deficits in the detection of contour-preserved and contour-violated differences, while lesions to left posterior temporal cortex were associated with selective impairments for the detection of differences where the contour was preserved. Taken together, this pattern of results suggests a model of hierarchical co-operativity whereby contour processing precedes interval processing and these two stages of the hierarchy are right and left lateralised in posterior superior temporal cortex. However, in a study similar to Peretz [5], Schuppert and colleagues [7] confirmed the notion of a processing hierarchy in patients with heterogenous cortical lesions, but the pattern of deficits did not support the proposed lateralisation of global–right; local–left.

The neuropsychological approach in patients with brain lesions is of clear value in establishing the necessity of brain areas for given functions. However, several aspects of the approach caution against a sole reliance on lesion data to derive neuro-anatomical models of cognitive processing. Brain lesions are rarely circumscribed, are heterogeneous across different patients, and may be functionally compensated for by other brain areas with a time-course that differs across patients. All these factors make assessment and interpretation of deficits challenging. Further, brain lesions occur within functional networks and particular damaged regions may not be sufficient in and of themselves to support the function, which may depend equally on other regions within a broader network. Functional imaging offers a valuable complement to the neuropsychological approach, providing a way to highlight the network of areas associated with the normal performance of a given function. The two approaches, when used in combination, provide a useful constraint on the interpretation of results and the formulation of new theories.

The present study used fMRI to test the model of Peretz and colleagues [5], [6] in neurologically normal individuals. The paradigm was modelled on the same/different tasks used in behavioural [2]–[4], [8] and patient studies [5], [6], but adapted to a one-back format which was more suited to fMRI. In addition, we used pitches that were drawn from a non-musical scale, so that findings could be generalized outside the purely musical domain [9], [10]. We tested the two predictions arising from the model: that the processing of pitch sequences involves a hierarchy (from a global to a local level) and that a different degree of lateralisation is seen for each of these stages (global–right; local–left). The results support the notion of a global to local processing hierarchy, as shown by greater activation for contour-preserved than contour-violated pitch sequences in right posterior superior temporal sulcus (pSTS) and planum temporale (PT). However, processing contour-violated sequences activated left pSTS, while contour-preserved sequences activated pSTS bilaterally, challenging the lateralisation scheme put forward by Peretz and colleagues [5], [6].

## Results

### Behavioural results

Twenty-four neurologically normal subjects underwent behavioural testing. We excluded four subjects who showed a difference in accuracy of more than 10% between the Local and Global conditions, to avoid confounding the interpretation of the imaging findings by differential performance between the Local and Global conditions. Mean correct performance in the scanner for these 20 subjects did not differ between Local (91.98%) and Global (93.15%) conditions (paired samples t-test, t_19_ = −1.17, p>0.1) and was significantly above chance (50%) (one-sample t-test, t_19_ = 28.07 and t_19_ = 39.12, both p<0.001, for Local and Global conditions, respectively).

### Effects of processing contour-preserved and contour-violated differences

**Figure 1:**
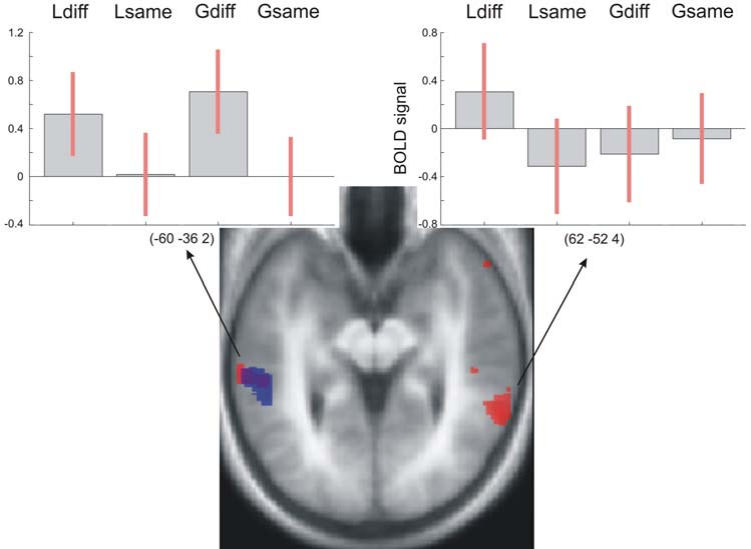
Activations for the *Local* ([Ldiff–Same]) (red) and *Global* ([Gdiff–Same]) (blue) contrasts superimposed on a tilted (pitch: −0.5) normalised average structural scan covering STS. Activations are thresholded at p<0.005 (uncorrected), for display purposes. Plots show the BOLD signal at local maxima in left and right pSTS. See also Figure S1.

Activation for Lsame and Gsame sequences did not differ from another, ruling out a potential ‘cognitive set’ effect, and we therefore pooled these two conditions as Same. In order to assess separately areas that are involved in the processing of local differences and global differences, we performed the following contrasts *Local*: ([Ldiff–Same]) and *Global*: ([Gdiff–Same]). *Local* revealed bilateral activation in pSTS, while *Global* was lateralised to the left pSTS, even at a reduced statistical threshold of p<0.05 (Figure 1; see also Figure S1 and Table 1).

**Table 1:** Stereotactic coordinates for the three contrasts *Local*, *Global*, and *Local–Global*.

Contrast	x	y	z	t-value
*Local* [Ldiff-Same]	62	−52	4	4.31
	−68	−36	−8	4.00
	−68	−40	−8	3.92
	−58	−36	0	3.65
*Global* [Gdiff-Same]	−60	−36	−2	3.94
	−54	−44	0	3.48
*Local-Global* [Ldiff-Gdiff]	68	−46	4	5.03
	60	−30	−2	4.98
	62	−22	8	4.75

In order to test whether the activation patterns for these contrasts (*Local* and *Global*) were significantly lateralised, we performed two formal tests of lateralisation using a routine within SPM5. The routine involves flipping the realigned and unwarped images about the anterior-posterior axis, and subsequently performing the normalisation, smoothing and statistical analysis procedures on these flipped images. To test for statistical differences between the left and right hemispheres for each contrast (*Local* and *Global*), a voxel-by-voxel pairwise t-test between the original and the flipped images was then performed.

These tests of lateralisation confirmed that no areas showed any lateralisation for *Local*, while *Global* was significantly lateralised to the left pSTS (Figure S2).

### Comparison of Local and Global Processing

**Figure 2:**
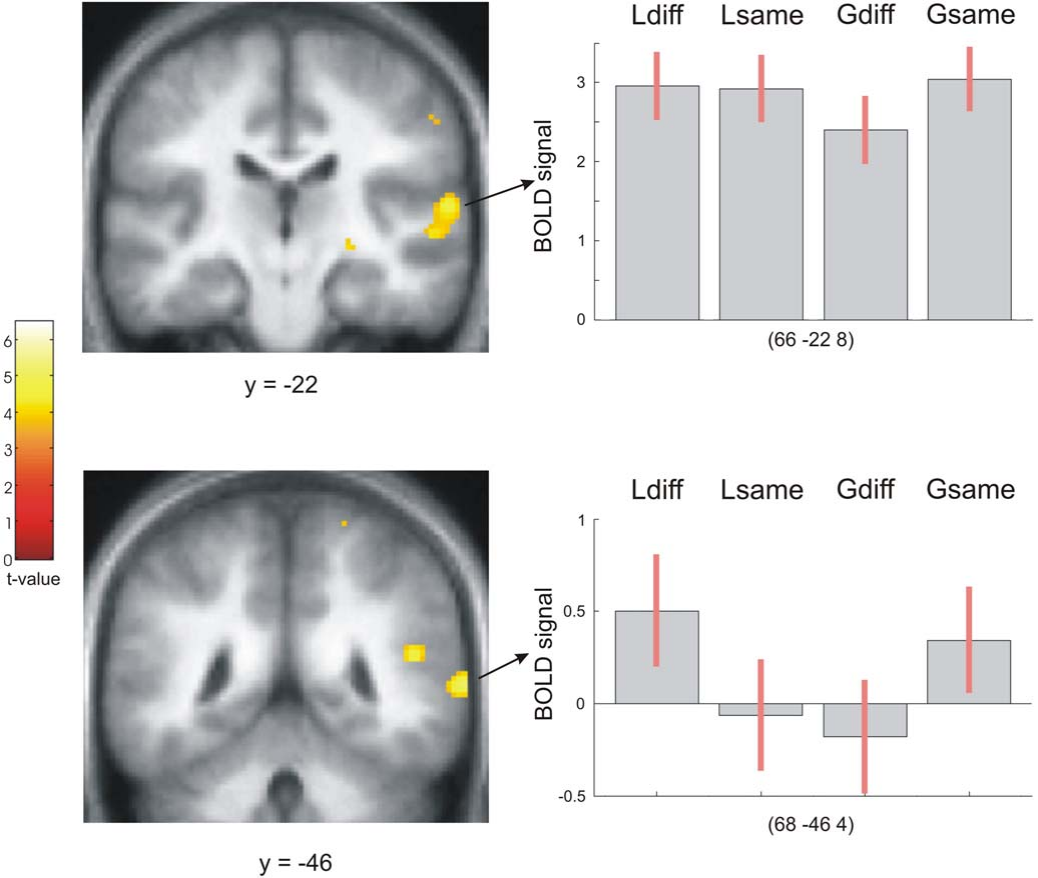
Activations for the *Local–Global* ([Ldiff–Gdiff]) contrast superimposed on coronal sections of participants' normalised average structural scan. Plots show the BOLD signal at local maxima in right PT (top right) and pSTS (bottom right).

We examined whether processing of local differences (contour-preserved) versus global differences (contour-violated) resulted in a distinct activation pattern via a contrast of *Local–Global* ([Ldiff–Gdiff]) and *Global–Local* ([Gdiff–Ldiff]). These directly compared activations corresponding to the detection of a contour-preserved difference versus a contour-violated difference and allowed us to test for a hierarchical relation between these two processes. *Local–Global* revealed activations in the pSTS and planum temporale (PT) on the right, while there were no significant differences for the *Global–Local* contrast (Figure 2, see also Table 1). A formal test of lateralisation confirmed these findings, showing right lateralised activations in pSTS and PT for *Local–Global* (Figure S3, see also Figure S1).

## Discussion

The aim of this study was to test both aspects of the model put forward by Peretz and colleagues [5], [6] which holds that the processing of pitch sequences involves a hierarchy (from global processing to local processing) and differential hemispheric lateralisation of these stages (global–right; local–left). The results of the present study confirm the hierarchy predicted by the model: a direct comparison of activation for the detection of a contour-preserved versus a contour-violated difference revealed greater activation for processing contour-preserved differences. No areas were more activated for processing of a contour-violated difference compared with a contour-preserved difference. The presence of additional activation for contour-preserved differences over and above those for contour-violated differences is consistent with a processing hierarchy in which local processing requires additional neural resources compared with global processing. However, our results contrast with the lateralisation account of the Peretz model [5], [6]: rather than demonstrating an association of global and local processing with the right and left hemispheres respectively, processing of change at the global level was lateralised to the left posterior STS, while processing of change at the local level engaged bilateral posterior STS. The location of these activations is congruent with results in Liégeois-Chauvel et al. [6], where damage to the posterior part of the superior temproal lobe (STL) was more detrimental for performance than anterior STL damage.

The processing hierarchy demonstrated here accords with cognitive neuropsychological and lesion-based evidence, and can be conceptualised as a fast serial search strategy whereby the first pitch sequence is encoded and provides a reference for the comparison of each of the constituent events of the second sequence. In such a scheme, incoming events are compared with the corresponding event in the first sequence, initially for contour direction (global) and then for the precise interval (local). If a difference is detected in contour, the search is terminated, otherwise the search process continues at the interval level. While the temporal resolution of fMRI is insufficient to provide direct support for this serial model, data including faster reaction times as well as earlier and greater event-related potentials to contour violations compared with contour-preserved differences provide strong evidence for such a serial search strategy [11]–[13].

Our findings concerning hemispheric lateralisation of local and global processing are at first more difficult to reconcile with lesion data and the predictions of the model by Peretz and colleagues which suggest a pattern of laterality such that local processing occurs within the left hemisphere and global processing within the right [5], [6]. However, a close examination of the neuropsychological studies urges a more circumspect interpretation. Two of these studies [5], [7] used unconventional cut-offs for defining impaired performance (the worst score and the mean score of the normal control (NC) groups, respectively), increasing the likelihood of false positive results. Further, in Peretz [5], although at a group level there was a pattern of deficits suggestive of a right–global; left–local dissociation, only five out of ten of the RHD patients had genuine global deficits (performance below cut-off), and only three out of ten of the LHD patients had genuine selective local deficits. Equally, in Liégeois-Chauvel [6], where lesion locations were confined to temporal cortex, three out of five patients with damage to right posterior temporal cortex had global deficits and one out of three patients with damage to left posterior temporal cortex had selective local deficits. Taken together, this more detailed picture suggest that the lateralisation scheme proposed by Peretz and colleagues [5], [6] can only partially account for the pattern of deficits observed in these patients.

We suggest that the processing scheme suggested by our data (global–left; local–bilateral) can account equally well for the pattern of results reported in previously published neuropsychological cases [5]–[7]. For example, in Liégeois-Chauvel [6], two out of five cases with right posterior temporal lesions showed either no deficit for local and global tasks or selective deficits in the local task alone, while two out of three patients with left posterior temporal cortex lesions were below cut-off for both local and global tasks. Furthermore, while LHD patients in Peretz [5] were better at global than local tasks, they nevertheless performed significantly worse than NC on both tasks.

While studies investigating local and global levels of auditory processing have generally confirmed the hierarchical account, evidence for hemispheric lateralisation of these levels has been more diverse and elusive [5]–[7], [11]–[14]. Clearly, further research using complementary experimental approaches and techniques is needed to refine the question of a lateralised hierarchy and to determine which parameters are relevant in driving the effect. In particular, there is a need for functional imaging studies of patients with focal brain lesions, to examine directly the distribution of processing following brain damage. Furthermore, various conceptualisations of local and global processing in the auditory domain are plausible [15]–[17], and it remains to be seen how these alternative formulations relate to one other. We further speculate that a left hemisphere ‘advantage’ for processing different levels of pitch pattern analysis (involvement of the left STS in both local and global processing, as shown here) may reflect specialisation for speech processing and the requirement for computation of both global and fine-grained pitch changes in the analysis of linguistic prosody [9], [10].

In conclusion, the present study is the first to demonstrate the neural bases of local and global levels of processing in pitch patterns in neurologically normal participants. The results show that local and global processing within pitch sequences differentially engage substrates in the posterior STS and that additional neural resources are required in the right posterior STS and PT for local pitch change processing. Our findings support the notion of a pitch pattern processing hierarchy that is likely to be generic rather than specific to music. Furthermore, the data suggest an alternative lateralisation scheme at these two levels of analysis which, while different to the traditionally held view, is equally consistent with the neuropsychological data from which this previous model is derived. The present study urges caution in accepting the traditional view of lateralisation, based on neuropsychological studies of local and global pitch sequence processing, and emphasizes the need for further research, both with patients and neurologically normal individuals, before an understanding of the functional lateralisation of local and global pitch sequence processing can be considered established.

## Materials and Methods

### Participants

Twenty four subjects were recruited for the study. All participants (10 male, 14 female) reported an absence of any hearing or neurological disorder and gave their informed consent. The experiment was carried out with the approval of the Institute of Neurology Ethics Committee, London and was in accordance with the ethical standards laid down in the 1964 declaration of Helsinki.

### Stimuli and Experimental Procedure

**Figure 3:**
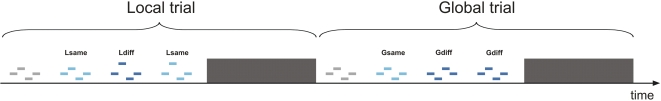
Schematic of two consecutive trials. Light blue indicates a pitch sequence that is identical to the previous sequence, dark blue indicates a pitch sequence that is different from the previous sequence; the first pitch sequence (grey) is neither the same nor different since there is no preceding pitch sequence. The scan period at the end of the trial is depicted in dark grey. Pitch sequences were 1200 ms long and separated by 800 ms gaps. Participants performed a one-back task, indicating whether a pitch sequence was same/different from the previous pitch sequence.

Since it was our intention to investigate local and global levels of auditory processing at a generic level, and not only in music, the pitches used were drawn from a set of frequencies that does not typically appear in combination in the Western musical tradition. Ten pitches, equally spaced in logarithmic steps, were taken from a two-octave range (120–480 Hz). Each pitch had a simple timbral envelope with a trapezoidal shape, 30 harmonics and a rise and decay time of 20 ms and 30 ms, respectively. Sounds were created digitally at a 44.1 kHz sampling rate and 16 bit resolution using Matlab (www.mathworks.com). A pitch sequence consisted of four 300 ms long pitches, amounting to a duration of 1.2 seconds per sequence. Each trial was made up of four pitch sequences separated by an inter-sequence interval of 800 ms. There were two experimental trial types: Local and Global (Figure 3). For both local and global trials, consecutive pitch sequences were the same (Lsame or Gsame) or different (Ldiff or Gdiff), with equal probability. In the Local trials, consecutive sequences, when different, had a pitch change at either the second or third element of the sequence with the constraint that this change did not alter the contour. Correct performance depended on perceiving a difference in the exact pitches or intervals in the two sequences. In the Global trials, consecutive sequences, when different, contained a pitch change brought about by reversing the order of the second and third elements, which always resulted in a difference in contour. Correct performance depended upon the perception of a difference in contour, in addition to any difference in the exact pitches or intervals in the two sequences. Participants pressed the key beneath their index and middle finger respectively, to indicate that the current sequence was the same or different to the previous. Participants were trained on each trial type outside the scanner, to a criterion level of 80%. During scanning, their performance was recorded and analyzed off-line for accuracy. In addition to Local and Global trials, there were also Silent trials comprising a period of silence matched to the duration of the other trial types. Participants performed two experimental sessions in which the three trial types: Local, Global and Silence were pseudo-randomly intermixed, with 64 instances for each of the two sessions.

### fMRI Protocol

Blood oxygen level-dependent (BOLD) contrast-image volumes were acquired on a 1.5 T scanner (Siemens MAGNETOM Sonata) using gradient echo planar imaging in a sparse protocol (repetition time 12.5 seconds), in order to temporally separate the scanner noise and the experimental sounds [18], [19]. A total of 48, 4 mm axial slices were acquired, with an in-plane resolution of 3×3 mm. One hundred and ninety-two brain volumes were acquired for each subject across the two sessions (64 for each condition). A high resolution T1 weighted structural image (1×1×1.5 mm) was also obtained. During scanning, stimuli were presented using Cogent (www.vislab.ucl.ac.uk/Cogent) and delivered via an external sound card (www.edirol.com) at a sound pressure level of 70 dB over a custom built electrostatic headphone system based on Koss™.

### Data Processing and Analysis

Imaging data were processed and analyzed using Statistical Parametric Mapping implemented with SPM5 software (www.fil.ion.ucl.ac.uk/spm). Scans from each subject were realigned to the first image of the time series and unwarped, spatially normalised to standard stereotactic space [20] and smoothed with an isotropic Gaussian kernel of 10 mm full-width-at-half-maximum.

Population-level inferences were made through a two-stage procedure. First, the data from each participant were analysed within the context of the general linear model [21]. Pitch sequences were categorised according to condition: Local Same (Lsame), Local Different (Ldiff), Global Same (Gsame) and Global Different (Gdiff) (Figure 3). Note that Lsame and Gsame sequences were identical and that the only difference was the context in which they were presented, either in a Local or a Global trial. Hence we modelled them separately to take account of potential ‘cognitive set’ effects. Each sequence was modelled as a short event of 1.2 seconds duration and was convolved with a haemodynamic response function. The first sequence of each trial was not modelled explicitly, since it was neither the same nor different. This approach explicitly models variance due to whether a given pitch sequence was same or different. From this model we derived parameter estimates for each condition. We used planned contrasts to assess differences in activation between the conditions, resulting in a contrast image. These contrast images were used in a second level random effects analysis. For each contrast of interest, we performed a one-sample t-test to derive statistical parametric maps (SPMs). Since we focus only on areas where we had an *a priori* prediction, i.e. in auditory cortex, we thresholded the SPMs at p<0.001 (uncorrected for multiple comparisons across the brain).

## Supporting Information

Figure S1 (0.71 MB DOC)

Figure S2 (0.06 MB DOC)

Figure S3 (0.04 MB DOC)
