# Supplementary material for: fMRI Evidence for a Cortical Hierarchy of Pitch Pattern Processing
Source: PLoS One. 2008 Jan 30;3(1):e1470. doi: 10.1371/journal.pone.0001470 (PMC2198945; doi:10.1371/journal.pone.0001470)
Supplement: Figure S1 — (0.71 MB DOC) [file pone.0001470.s001.doc]

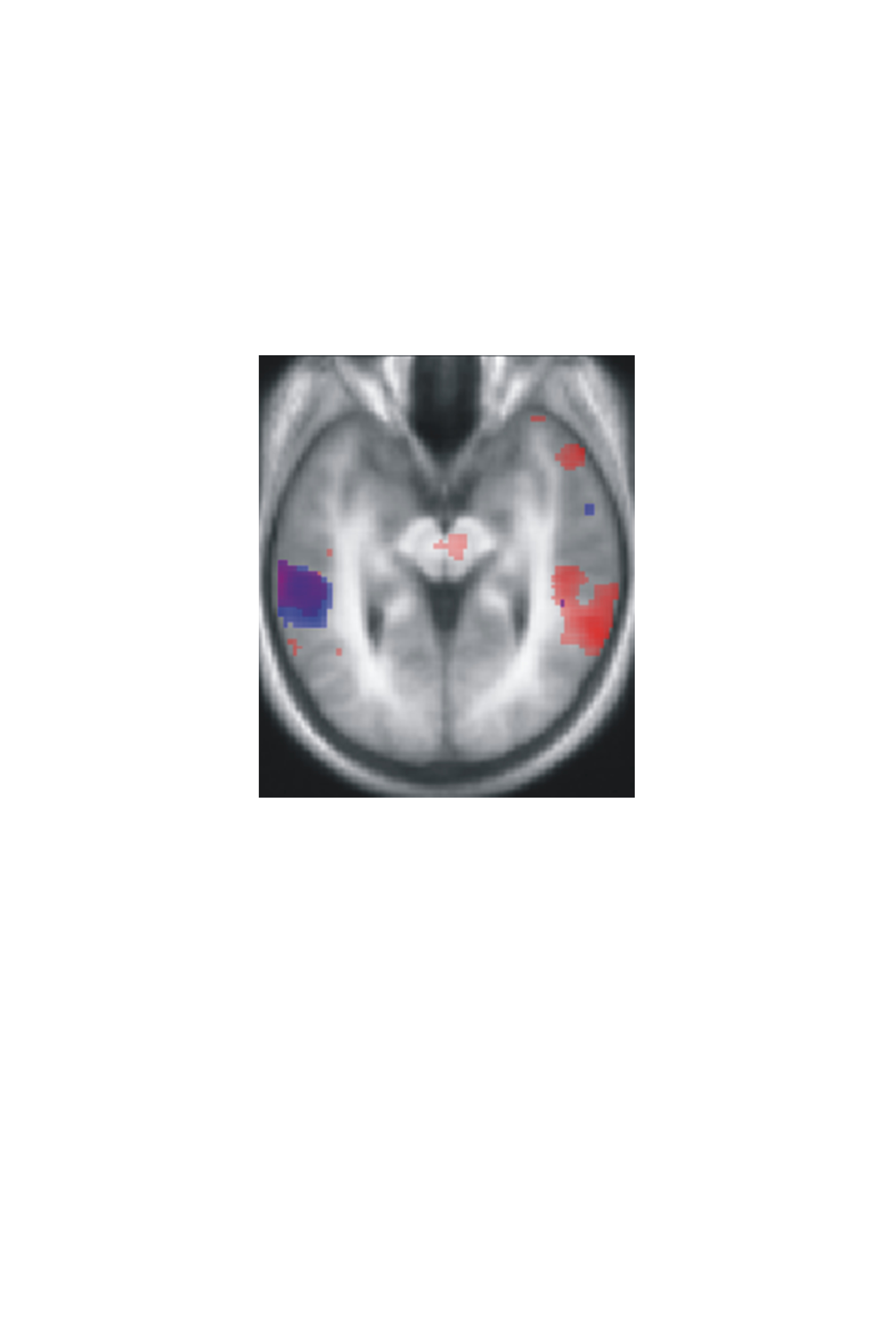


**Figure S1**

Same as Figure 2, thresholded at p < 0.05, uncorrected for multiple comparisons. Note that *Global* does not show any activation in right pSTS.
