# Supplementary material for: fMRI Evidence for a Cortical Hierarchy of Pitch Pattern Processing
Source: PLoS One. 2008 Jan 30;3(1):e1470. doi: 10.1371/journal.pone.0001470 (PMC2198945; doi:10.1371/journal.pone.0001470)
Supplement: Figure S2 — (0.06 MB DOC) [file pone.0001470.s002.doc]

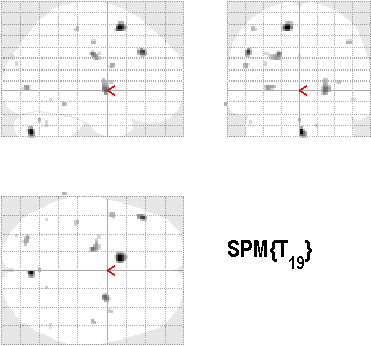


**(a)**


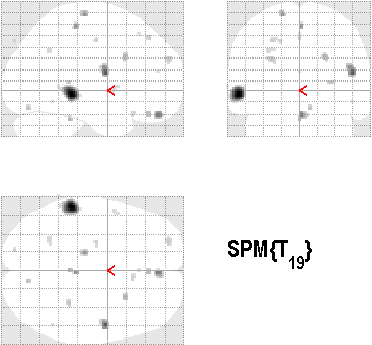


**Figure S2**

Results for the lateralisation test of **(a)** *Local* ([Ldiff – Same]) for original – flipped scans, and **(b)** *Global* ([Gdiff – Same]) for original – flipped scans.

**(b)**
