# Supplementary material for: fMRI Evidence for a Cortical Hierarchy of Pitch Pattern Processing
Source: PLoS One. 2008 Jan 30;3(1):e1470. doi: 10.1371/journal.pone.0001470 (PMC2198945; doi:10.1371/journal.pone.0001470)
Supplement: Figure S3 — (0.04 MB DOC) [file pone.0001470.s003.doc]

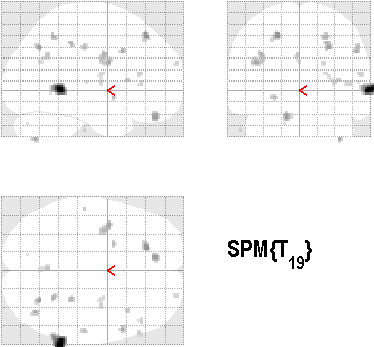


**Figure S3**

Results for the lateralisation test of *Local - Global* ([Ldiff – Gdiff]) for original – flipped scans.
